# Significant Roles of Regulatory T Cells and Myeloid Derived Suppressor Cells in Hepatitis B Virus Persistent Infection and Hepatitis B Virus-Related HCCs

**DOI:** 10.3390/ijms16023307

**Published:** 2015-02-03

**Authors:** Yasuteru Kondo, Tooru Shimosegawa

**Affiliations:** Division of Gastroenterology, Tohoku University Graduate School of Medicine 1-1 Seiryo, Aoba, Sendai City, Miyagi 980-8574, Japan; E-Mail: tshimosegawa@int3.med.tohoku.ac.jp

**Keywords:** regulatory T cells (Tregs), myeloid derived suppressor cells (MDSCs), hepatitis B virus (HBV), hepatocellular carcinoma (HCC)

## Abstract

The adaptive immune system, including type1 helper T cells (Th1 cells), cytotoxic T lymphocytes (CTLs), and dendritic cells (DCs), plays an important role in the control of hepatitis B virus (HBV). On the other hand, regulatory T cells (Tregs) and myeloid derived suppressor cells (MDSCs) suppress the immune reaction in HBV and hepatocellular carcinoma (HCC). Excessive activation of immune suppressive cells could contribute to the persistent infection of HBV and the progression of HCC. The frequency and/or function of Tregs could affect the natural course in chronic hepatitis B patients and the treatment response. In addition to the suppressive function of MDSCs, MDSCs could affect the induction and function of Tregs. Therefore, we should understand in detail the mechanism by which Tregs and MDSCs are induced to control HBV persistent infection and HBV-related HCC. Immune suppressive cells, including Tregs and MDSCs, contribute to the difficulty in inducing an effective immune response for HBV persistent infection and HBV-related HCC. In this review, we focus on the Tregs and MDSCs that could be potential targets for immune therapy of chronic hepatitis B and HBV-related HCC.

## 1. Introduction

Hepatitis B virus (HBV) is a noncytopathic DNA virus that causes chronic hepatitis and hepatocellular carcinoma (HCC) as well as acute hepatitis [1]. HBV infects more than 400 million people worldwide. HBV has six different genotypes [2,3]. The progression of liver fibrosis and HCC could vary among various HBV genotypes [3,4].

It has been reported that the innate immune system, including intra-hepatocyte reactions [5,6], natural killer cells (NK cells), natural killer T cells (NK-T cells), and monocytes, could contribute to the immunopathogenesis of HBV infection [7,8,9,10,11,12]. However, the adaptive immune system, including type 1 helper T cells (Th1 cells), cytotoxic T lymphocytes (CTLs), and dendritic cells (DCs), plays an important role in the control of HBV [13,14,15,16,17,18]. On the other hand, CD4^+^CD25^+^FOXP3^+^ regulatory T cells (Tregs) and myeloid derived suppressor cells (MDSCs) suppress the immune reactions to HBV and HCC [19,20,21,22,23,24]. The excessive activation of immune suppressive cells could contribute to the persistent infection of HBV and the progression of HCC. Tregs constitutively express CD25 in the physiological state [25]. In humans, this population, as defined by CD4^+^CD25^+^FOXP3^+^ T cells, constitutes 5% to 10% of peripheral CD4^+^ T lymphocytes and has a broad repertoire that recognizes various self and non-self antigens [26,27,28]. Various kinds of immune suppressing mechanisms-induced by Tregs have been reported [28]. The important mechanisms are cell-to-cell contact and secretion of immune-suppressive cytokines including transforming growth factor-β (TGF-β) and IL10 [19,23,28]. An emerging cell population of interest, MDSCs, could contribute to immune suppression. MDSCs are a heterogeneous population of immature myeloid cells, originally shown to accumulate at the sites of tumors. MDSCs have been well described in various severe human diseases such as cancer, autoimmune diseases, and bacterial infections [19,29,30]. In mouse, populations of MDSCs have been divided into two groups: polymorphonuclear MDSCs (PMN-MDSC), described as CD11b^+^Gr-1^high^Ly6^+^Ly6C^low/int^ cells, and mononuclear MDSCs (Mo-MDSC), described as CD11b^+^Gr-1^int^Ly6G^−^Ly6C^high^ cells [31,32]. On the other hand, in human, the phenotypic markers of MDSCs are less clear. MDSCs have been described as CD33^+^CD11b^+^HLA-DR^l^^o^^w/−^ in a human cancer model [32] (Table 1). It has been reported that MDSCs could contribute to persistent infection of HBV and HCC [19,20,30,33,34,35,36,37,38] (Table 1). In addition to HBV and HCC, MDSCs could contribute to persistent infection of HCV [39,40]. HCV core-treated CD33^+^ cells exhibit a CD14^+^CD11b^+/low^ HLA-DR^−/low^ phenotype with up-regulated reactive oxygen species (ROS) production [39]. Moreover, the frequency of MDSCs increases and correlates with HCV-RNA loads in chronic hepatitis C (CH-C) patients [40]. MDSCs could suppress the T cell response by way of numerous mechanisms including the expression of inhibitory cell surface molecules, the production of immune suppressive cytokines, the metabolism of arginine through activation of arginase-1, production of nitric oxide, and the up-regulation of reactive oxygen species. Moreover, an interaction between MDSCs and Tregs has been reported [20,31,32,41,42,43]. MDSCs and Tregs could contribute to not only HCC but also various kinds of cancers [43,44,45,46,47,48,49,50,51,52,53,54,55,56,57,58,59,60,61,62,63] (Table 2). Many groups reported the frequency of MDSCs, and Tregs could be useful prognostic biomarkers. Moreover, some reports indicated the possibility of modifying the function of MDSCs and Tregs to improve the immune reaction to cancers by using CTLA-4 antibody, PD-L1 antibody, anti-miR214, c-kit antibody and sunitinib, *etc.* (Table 2).

**Table 1:** Association between MDSCs and HBV or HCC.

Species	MDSCs Phenotype	Diseases or Models of Diseases	Functions and Findings	References
Human	Lin-HLA-DR-CD11b^+^CD33^+^	HBV	MDSCs might be involved in HBeAg immune tolerance	Lu *et al.* [30]
Mouse	CD11b^+^Gr1^+^	HBV (mouse model)	gammadelta T Cells drive MDSCs–mediated CD8+ T Cell exhaustion	Kong *et al.* [33]
Mouse	CD11b^+^Gr1^+^	HBV (mouse model)	In HBV TM, the frequencies of liver MDSCs were about twice those of normal mice liver	Chen *et al.* [34]
Human	CD34^+^CD14^−^HLA-DR^−^CD11b^+^	HBV vaccination in HIV patients	High frequency of MDSCs contribute to week 16 HBV vaccine response	Anthony *et al.* [35]
Human	CD14^+^HLA-DR^−^^/low^	HCC	MDSCs induces CD4+CD25+Foxp3+ T Cells Frequency of CD14+ HLA-DR-/low cells Is Increased in PBMC and tumor of HCC patients	Hoechest *et al.* [19]
Human	CD14^+^HLA-DR^−^^/low^	HCC	MDSCs inhibit NK cells in HCC patients via the NKp30 receptor	Hoechest *et al.* [36]
Human	CD14^−^HLA-DR^−^CD11b^+^CD33^+^	HCC	Elevated numbers of MDSC in HCC patients Over-production of inhibitory cytokines such as IL10 and TGF-β	Kalathil *et al.* [20]
Mouse	Gr^+^CD11b^+^	HCC mouse model	An accumulation of MDSC is found in various mice models with HCC	Kapanadze *et al.* [37]
Mouse	Gr^+^CD11b^int^	HCC mouse model	Tumors produce IL-6 and VEGF and induced iMC (CD11b1Gr-1int)	Shmidl *et al.* [38]

**Table 2:** Association of Tregs and MDSCs and other cancers except for HCC.

Cancer	Immune Suppressive Cells		Treatments, Future Treatments or Other Uses	References
	Tregs	MDSCs		
Large B cell lymphoma	○		prognostic biomarker	Ahearne *et al.* [44]
Non-Hodgkin Lymphoma	○		prognostic biomarker	Pinheiro *et al.* [45]
Myeloma	○		CTLA-4 antibody	Braga *et al.* [46]
	○	○	High-dose IL2 followed by Sorafenib	Monk *et al.* [47]
		○	Adjuvant GM-CSF	Daud *et al.* [48]
	○	○	prognostic marker	Brimnes *et al.* [49]
Ovarian Cancer	○		prognostic biomarker	Brtnicky *et al.* [50]
Mouse model (liver metastasis model)		○	Anti-PD-L1 antibody	Ilkovitch *et al.* [51]
Esophageal Cancer	○		Down-regulation of B7-H1 expression	Chen *et al.* [52]
non-small-cell lung cancer	○		prognostic biomarker	Hasegawa *et al.* [53]
				Yukawa *et al.* [54]
		○	prognostic biomarker	Liu *et al.* [43]
Lewis lung carcinoma (mouse model)	○		anti-miR214	Yin *et al.* [55]
Pancreatic ductal adenocarcinoma	○		prognostic biomarker	Luardi *et al.* [57]
Renal Cell Carcionam	○	○	High-dose IL2 followed by Sorafenib	Monk *et al.* [47]
		○	prognostic biomarker	Mirza *et al.* [58]
		○	prognostic biomarker	Kusmartsev *et al.* [59]
Solitary Fibrous Tumor		○	Sunitinib malate	Tazzari *et al.* [60]
Thyroid cancer	○		Inhibition of FOXP3	Chu *et al.* [61]
Glioblastoma	○		prognostic marker	Soyour E *et al.* [62]
Colon carcinoma (mouse model)	○	○	c-kit antibody	Pan *et al.* [63]

Recently, nucleoside analogues have emerged as an important treatment option for chronic hepatitis B patients [64]. However, discontinuation of nucleoside analogues could frequently induce the reactivation of chronic hepatitis [65,66]. Therefore, pegylated interferon (Peg-IFN) could be a significant treatment to control replication of HBV by inducing an immune response. The efficacy of Peg-IFN treatment has not yet become optimal [67]. Immune suppressive cells including Tregs and MDSCs might contribute to the difficulty of inducing an effective immune response for HBV persistent infection and HCC. This review focuses on the Tregs and MDSCs that could be potential targets for the immune therapy of chronic hepatitis B patients and HCC.

## 2. Tregs Could Affect HBV Persistent Infection

In addition to hepatitis c virus infection (HCV), it has been reported that the HBV-specific immune response could be suppressed by CD4^+^CD25^+^ Tregs in patients with HBV infection [68]. This report indicated that not only Tregs from CH-B patients, but also those from patients with resolved HBV infection could suppress HBV specific CD8^+^ T cell. However, it has been reported that the frequency of Tregs in CH-B patients was significantly higher than those in healthy controls and those with resolved HBV infection [69]. Therefore, the frequency of Tregs might contribute to the disease status of HBV infection [23,24,68,69,70,71,72,73,74,75,76] (Table 3). Tregs have been identified by using CD4, CD25, CD45RO and CTLA-4 antibodies. Another group reported that the frequency of CD39^+^Tregs correlates with the progression of HBV infection [77]. Therefore, we should consider this minor subset of Tregs in chronic hepatitis B patients [77]. Previously, we reported that HBcAg-specific IL10 secreting CD4^+^CD25^+^ Tregs might contribute to the suppression of HBV-specific IFN-gamma secreting CD4^+^ T cells [23]. Moreover, the depletion of Tregs could recover the function of IFN-gamma secretion of CD4^+^ T cells in an *ex vivo* study [23]. Another group reported a similar phenomenon and the enhancement of HBV-specific T cell proliferation after the depletion of Tregs [69]. Previously, several groups including ours reported that the reduction of HBV could recover the frequency of HBV-specific T cells and the function of T cells [15,78]. Tregs might contribute to suppressing the HBV-specific T cells. Therefore, treatment with a nucleos(t)ide analogue might affect the Tregs. Previously, Stoop *et al.* [71] described that adefovir-induced viral load reduction caused a decrease in circulating Tregs together with a partial recovery of the immune response. They described that the frequency of Tregs among CD4^+^ T cells was decreased at three and six months after adefovir treatment. Moreover, they determined that the frequency of HBcAg-specific IFN-gamma secreting cells was increased during adefovir treatment. We also reported that the entecavir-induced viral load reduction caused a decrease in circulating Tregs [24]. Moreover, we analyzed the mechanism of Tregs enhancement of functions in chronic hepatitis B patients [24]. Heat shock protein 60 produced from HBV-replicative hepatocytes might enhance the IL 10-secreting function of Tregs via toll like receptor 2 (TLR2). The inhibition of TLR2 signaling could inhibit the excessive function of Tregs. Another group reported that over-expression of TLR2/4 on monocytes could modulate the activities of Tregs in chronic hepatitis B patients [79]. This report described that the agonists of TLR2 and 4 activated-Tregs showed enhanced suppression function in chronic hepatitis B patients. Another group reported that exogenous tumor necrosis factor alpha partially abrogated the Tregs-mediated suppression [80]. The interaction between programmed death (PD)-1 and its ligand, PD-L1, is important for the induction of exhausted T cells. In chronic hepatitis B patients, the antiviral intrahepatic T cell response could be restored by blocking the PD-1 pathway [81]. Tregs express both PD-L1 and PD-1 [82]. That PD-L1/PD-1 signaling might suppress the HBV-specific immune response has been reported by many groups [81,83,84,85,86]. The inhibition of PD-1 and cytotoxic lymphocyte antigen-4 (CTLA-4) could slightly enhance the cellular proliferation and significantly increased the IFN-gamma production of PBMCs co-cultured with Tregs [84]. Concerning the CD4^+^ development pathway, both induced Tregs and Th17 cells require TGF-β. In addition to TGF-β, IL-2 promotes development of Tregs and inhibits Th17 cells, whereas IL6, IL21 and IL23 promote the development of Th17 cells and inhibit that of Tregs. Therefore, the balance between Tregs and Th17 cells during hepatitis B virus infection was analyzed [87,88,89,90]. A group reported that acute or chronic HBV-related liver failure patients have a dramatically higher IL17^+^/FOXP3^+^ ratio than that in chronic liver failure patients [87]. Another report described that a lower Treg/Th17 ratio induced greater liver fibrosis progression [88]. Although these findings are unsurprising, the balance between Tregs and Th17 might be usefully analyzed for the disease status and treatment response. A group described that the frequency of Tregs increased in non-responders but not in responders during pegylated-interferon [91]. The frequency and/or function of Tregs could affect the natural course of chronic hepatitis B patients and treatment response [92]. Most of the groups analyzed the peripheral blood to detect Tregs (Table 3). Some groups analyzed liver-infiltrating lymphocytes in addition to peripheral blood to detect Tregs. Xu *et al.* [70] indicated that the frequency of liver-infiltrating Tregs increased in CH-B patients and chronic severe hepatitis B patients, as seen in peripheral blood. Yang *et al.* [72] reported that Foxp3+ cells were present in significantly higher numbers in liver tissue sections from chronic active hepatitis B, as seen in peripheral blood. However, it is difficult to carry out the sequential analysis of liver-infiltrating lymphocytes since liver biopsy has a risk of bleeding.

**Table 3:** Association between HBV infection and Tregs.

Species or Model	Disease Status	Immune Subset	Frequency, Functions or Findings	References
Human	AHB Recovered	Isolated CD4^+^CD25^+^	Suppression of CD8^+^ cells	Franzese *et al.* [68]
	CHB		Frequecny (AHB = CHB = Healthy)	
	Healhty subjects			
Human	AHB Recovered	Isolated CD4^+^CD25^+^	Suppression of CD4^+^ cells	Stoop *et al.* [69]
	CH-B		Frequency(Chronic > recovered: Chronic > healthy donors)	
	Healthy subjects			
Human	AH-B	Isolated CD4^+^CD25^+^; FOXP3^+^ gated liver infiltrating lymphocytes	Suppression of CD4^+^ cells	Xu *et al.* [70]
	CH-B		Frequency(CH-B severe > CHB, CHB severe > AH-B, CH-B severe > healthy donors)	
	Healthy subjects			
Human	CH-B	Isolated CD4^+^ CD25^+^	Suppression of CD4^+^ cells	Kondo *et al.* [23]
	Healthy subjects		Chronic = healthy donors	
Human	Treated CH-B	CD4^+^ CD25^+^ CTLA4^+^ CD45RO^+^ (FOXP3^+^)	Frequency (Treated CHB < CHB)	Stoop *et al.* [71]
	CH-B			
	Healthy subjects			
Human	Recovered AH-B	Isolated CD4^+^CD25^+^; FOXP3^+^ liver infiltrating lymphocytes	Suppression of CD4^+^ cells and CD8^+^ cells	Yang *et al.* [72]
	CH-B		Frequency(Chronic asymptomatic > chronic active > resolved = healthy controls)	
	Healthy subjects			
Human	CH-B	CD4^+^CD25^+^IL7R^-^	sHSP60 enhances Tregs activity via TLR2 signaling	Kondo *et al.* [24]
	Treated CH-B patients		Frequency (Treated CHB < CHB)	
	Healthy subjects			
Woodchuck hepatits	Woodchuck hepatitis	CD4^+^FOXP3^+^	Frequency (WHV > Control)	Otano *et al.* [73]
HBV model			Interleukin-12 Increases Hepatic Tolerogenicity	
Mouse	AdHBV	CD4^+^FOXP3^+^	Down-regulating the antiviral activity of effector T cells by limiting cytokine production and cytotoxicity	Stross *et al.* [74]
Human	HBV-HCC	CD4^+^CD25^+^FOXP3	TGF-b-miR-34a-CCL22 Signaling-Induced Treg Cell Recruitment	Yang *et al.* [75]
Human	CH-B	CD4^+^CD25^+^	Frequency (CH-B = Acute on choronic HBV = Healthy)	Dong *et al.* [76]
	Acute on choric HBV			
	Healthy			

## 3. Tregs and HBV-Related HCC

It has been reported that the frequency of Tregs is increased in HCC patients [93,94]. Yang *et al.* [93] analyzed the frequency of Tregs and CD8^+^ T cell in peripheral blood and liver tissue. The results indicated a significant increase in both the proportion and absolute numbers of CD4^+^CD25^+^ T-cells in the peri-tumor region [94]. Another group indicated the higher frequency of Tregs in the peripheral blood from HCC patients in comparison to those from HCV patients and healthy subjects. The mechanisms of increased Tregs in HCC were analyzed. Huh7 culture supernatants appear to promote CD4^+^CD25^+^ T-cell proliferation and inhibit CD4^+^CD25^−^ T-cell proliferation [95]. Moreover, the frequency of Tregs could be a significant biomarker of survival in HCC patients. The frequency of circulating CD4^+^CD25^+^FoxP3^+^ Tregs was increased significantly and correlated with the disease progression in HBV-related HCC patients [22,96,97]. An abundant accumulation of Tregs concurrent with a significantly reduced infiltration of CD8^+^ T cells was found in tumor regions compared with nontumor regions [96]. Another group reported that the frequency of the CD45RO^+^ subset in CD4^+^CD25^high^ Tregs was associated with progression of HCCs [98]. Moreover, the frequency of the other phenotype of Tregs (CD4^+^CD25^−^CD69^+^) was also increased in HCC patients [99]. The induction of Tregs could be affected by not only hepatitis B virus infection but also HCC. When PBMCs were co-cultured with human hepatoma cell lines stably transfected with HBV (HepG2.2.15), the CD4^+^CD25^+^ Tregs population increased and upregulated Tregs-related genes [100]. Sorafenib is a multikinase inhibitor that could suppress cell proliferation and angiogenesis. Sorafenib could reduce the frequency of hepatic infiltrating Tregs by suppressing TGF-β signaling [101]. Suppressing Tregs might be one of the significant targets for the induction of immunity for HCC.

## 4. MDSCs for HBV Persistent Infection and HBV-Related HCC

An emerging cell population of interest, MDSCs, could contribute to immune suppression. In a mouse model, it has been reported that liver-derived MDSCs from HBV transgenic mice could suppress the proliferative capacities of allogenic T cells and HBsAg-specific lymphocytes [34]. Recently, it has been reported that γδT cells could drive MDSCs-mediated CD8^+^ T cell exhaustion in HBV persistent infection [33]. In addition to the suppressive function of MDSCs, MDSCs could affect the induction and function of Tregs [19]. It has been reported that MDSCs could induce Tregs in HCC patients [19]. Moreover, another group reported that higher frequencies of GARP^+^CTLA-4^+^Foxp3^+^ Tregs and MDSCs in HCC patients are associated with impaired T-cell functionality [20]. Compared to Tregs, few reports have described the relationship between MDSCs and HCC. However, many groups including ours are focusing on MDSCs for the induction-mechanism of Tregs in HBV persistent infection and HBV-related HCC.

## 5. Concluding Remarks

Many groups including ours have reported that Tregs and MDSCs suppress the immune reaction for HBV and HCC. The excessive activation of immune suppressive cells could contribute to the persistent infection of HBV and the progression of HCC. Therefore, detailed mechanisms of the induction of Tregs and MDSCs should be investigated to control HBV persistent infection and HBV-related HCC. Moreover, the ability to specifically suppress Tregs and MDSCs, and understanding the appropriate time point to do so, might improve the treatment of HBV-related diseases. 
